# Validation of Plantar Pressure and Reaction Force Measured by Moticon Pressure Sensor Insoles on a Concept2 Rowing Ergometer

**DOI:** 10.3390/s21072418

**Published:** 2021-04-01

**Authors:** Georgina Kate Barratt, Clint Bellenger, Eileen Yule Robertson, Jason Lane, Robert George Crowther

**Affiliations:** 1 UniSA: Allied Health and Human Performance, University of South Australia, 5001 Adelaide, Australia; clint.bellenger@unisa.edu.au (C.B.); Robert.Crowther@unisa.edu.au (R.G.C.); 2 Alliance for Research in Exercise, Nutrition & Activity (ARENA), University of South Australia, 5001 Adelaide, Australia; 3 South Australian Sports Institute, 5025 Adelaide, Australia; eileen.robertson@sa.gov.au (E.Y.R.); jason.lane@sa.gov.au (J.L.)

**Keywords:** force, pressure, biomechanics, Moticon, Pedar-x, rowing

## Abstract

The purpose of this study was to determine the reliability and validity of plantar pressure and reaction force measured using the Moticon and Pedar-x sensor insoles while rowing on a Concept2 ergometer. Nineteen participants performed four 500 m trials of ergometer rowing at 22–24 strokes/min; two trials wearing Moticon insoles and two wearing Pedar-x insoles in a randomised order. Moticon and Pedar-x insoles both showed moderate to strong test–retest reliability (ICC = 0.57–0.92) for mean and peak plantar pressure and reaction force. Paired t-test demonstrated a significant difference (*p* < 0.001) between Moticon and Pedar-x insoles, effect size showed a large bias (ES > 1.13), and Pearson’s correlation (r < 0.37) showed poor agreement for all plantar pressure and reaction force variables. Compared to Pedar-x, the Moticon insoles demonstrated poor validity, however, the Moticon insoles had strong reliability. Due to poor validity, caution should be used when considering Moticon insoles to assess changes in pressure and force reliably over time, across multiple trials or sessions. Moticon’s wireless and user-friendly application would be beneficial for assessing and monitoring biomechanical parameters in rowing if validity between measures of interest and Moticon’s results can be established.

## 1. Introduction

Sensor insoles have been used to determine plantar pressure and reaction force data in sports performance analysis [1], foot-wear design [2], injury prevention [3], rehabilitation [4], clinical gait analysis [5], and balance control [6,7]. A number of insole systems exist, most notably Pedar-x (Novel GmbH, Munich, Germany) and Moticon (Moticon ReGo AG, Munich, Germany). The Pedar-x in-shoe insole system has been shown to reliably measure plantar pressure variables (coefficient of variation [CV] = 8.8–22.5%), force variables (CV = 7.7–32.2%), and temporal-spatial variables (CV = 4.7–17.6%) during walking gait [8], with a coefficient of repeatability (CR) [9] of less than 15.3% for pressure and temporal parameters in running [10]. In addition, Pedar-x system has been shown to be valid (Root mean square error (RMSE) 2.6 kPa; difference 3.9%) for pressure variables when tested using a TruBlue device to apply and even load over the insole surface at a range of pressures (50, 100, 200, 300, 400, 500 and 600 kPa) [11]. Pedar-x insoles measurement of the vertical force during walking gait (the second peak force occurring at the toe-off phase) was valid (*p* > 0.546) when compared to a force platform, however, after long term use (>3 h) drift was found to occur [12]. A wireless insole may be more practical than a wired system for research and routine monitoring in applied settings, allowing athletes to perform comfortably and naturally. The Moticon insole system is a wireless, in-shoe system with good practical application due to its minimally invasive set-up. The reliability and validity of Moticon insoles for quantifying plantar pressure, reaction force, centre of pressure (COP) and gait temporal-spatial parameters has been assessed during walking and running [13,14,15], jumps [15], and balance tasks [15,16]. Specifically, Braun et al. [13] found strong test–retest reliability for all measured force and temporal gait parameters (intraclass correlation coefficient [ICC] > 0.983). This was supported by Oerbekke et al. [16] who determined excellent reliability for temporal gait parameters (ICC_2_,_1_ = 0.90–0.93).

Force variables calculated by Moticon have been compared to force platforms [14,15,16], force plate instrumented treadmills [13], and the Pedar-x insole sensor system [15]. Discrepancies in results are present when evaluated across different movements and activities, due to variations in speed and applied force. In walking gait (1.0 m/s and 1.7 m/s), Braun et al. [13] found no difference for resultant force (*p* = 0.19) and stance time (*p* = 0.36) between Moticon and the Zebris pressure platform (Zebris Medical GmbH). Analysis of bias and 95% limits of agreement (LoA) in recent studies comparing Moticon to Pedar-x insole sensor system, force platforms, and force plate instrumented treadmills, indicate differences in force variables and temporal-spatial parameters [14,15,16].

Agreement between force measuring systems was greater when ground contact times were longer and the applied forces were lower (e.g., during walking) [15]. In activities with a short ground contact time and high applied force (e.g., sprinting and jumping), Moticon was found to underestimate force variables [14,15,16]. However, despite underestimation of force variables, they were found to be highly correlated (r = 0.56–0.93) with Pedar-x insoles and force platforms, indicating good relative agreement [14,15]. High correlations between Moticon insoles and force platforms demonstrated clear heteroscedasticities, indicating that as the applied force increased, the magnitude of the underestimation by Moticon insoles also increased [14].

Rowing is a sport heavily influenced by biomechanical and physiological factors [17]. The lower limbs apply a pushing force against the foot-stretcher (the platform where the rower’s feet are placed in the boat), to accelerate the rower’s centre of mass, resulting in the acceleration of the boat as the oars are pulled through the water. While force at the oarlock can be routinely measured by various commercial systems, forces at the foot-stretcher should be analysed concurrently to provide a more comprehensive understanding of rowing performance [18]. Studies investigating foot-stretcher force profiles have often used custom-built constructions including load cells, strain gauges, and transducers on ergometers [19,20,21,22,23,24] and in rowing boats [25,26]. These custom-built methods are not accessible for many rowing programs and coaches, as they can be expensive, time-consuming to set-up and analyse, and may negatively impact boat and athlete dynamics. Consequently, current literature lacks experimental research exploring foot-stretcher force profiles during on-water rowing [27]. Owing to their cables, wires, and additional devices for data storage and battery power, the Pedar-x insoles are not practical for on-water rowing, and as such the wireless set-up of the Moticon insoles potentially makes them a feasible, cost-effective, time-efficient, and a highly mobile application for measuring plantar pressure and reaction force variables. However, the validity and reliability of Moticon insoles has not been investigated in rowing.

Consequently, the aim of this study was to determine the reliability and validity of plantar pressure and reaction force measured by Moticon sensor insoles in comparison to PedarX sensor insoles on a Concept2 rowing ergometer. It was hypothesised that (1) the Moticon and Pedar-x insoles will display moderate to strong test–retest reliability (ICC > 0.6) for plantar pressure and force variables, and (2) that there would not be a significant difference (*p* > 0.05) between the Moticon and Pedar-x insoles for pressure and force variables, however, there would be bias (>30%) towards Pedar-x insoles as a result of the larger surface area covered by the sensors in Pedar-x insoles.

## 2. Materials and Methods

### 2.1. Participants

Nineteen rowers (female = 16, male = 3; age (mean ± SD), 18.6 ± 0.5 years; height, 1.70 ± 0.04 m; body mass, 67.0 ± 6.9 kg) were recruited from rowing programs within South Australia, Australia. Inclusion criteria for participants were aged 16 years or older, a minimum of three years of rowing experience (including experience using a Concept2 rowing ergometer), and free of any neuromusculoskeletal injuries. Ethics approval was granted from the University Human Research Ethics Committee (202249) and written consent was obtained from all participants prior to testing.

### 2.2. Experimental Overview

**Figure 1:**
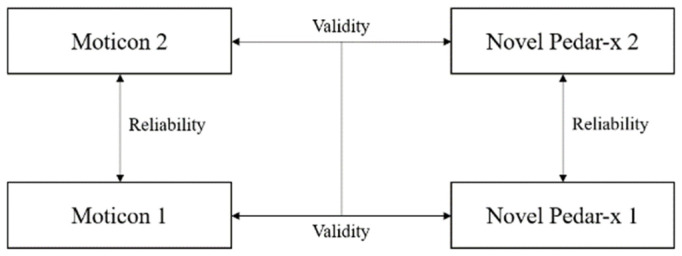
Randomised crossover design for assessing the reliability and validity of the Moticon sensor insoles.

The study was a randomised crossover design. Participants were tested using two different insole pressure-measuring systems: Moticon (SensorInsole2, Moticon ReGo AG, Munich, Germany) and Novel Pedar-x (Pedar, Novel GmbH, Munich, Germany), on a Concept2 modelD stationary rowing ergometer (Concept2 Inc., Morristown, VT, USA) with PM5 display. A mobile application Float Pro (2.1.11 (5), Float, Endurance Sports Research Ltd, Cambridge, UK) was connected to the ergometer PM5 display by Bluetooth to collect performance variable data. Participants attended the laboratory for one session where they performed four 500 m time trials in a randomised order. Each set of insoles was worn on two trials to assess test–retest reliability, and the pooled data from the two trials of the same insoles were used to compare the Moticon to the Pedar-x for validity (Figure 1).

### 2.3. Instrumentation

**Figure 2:**
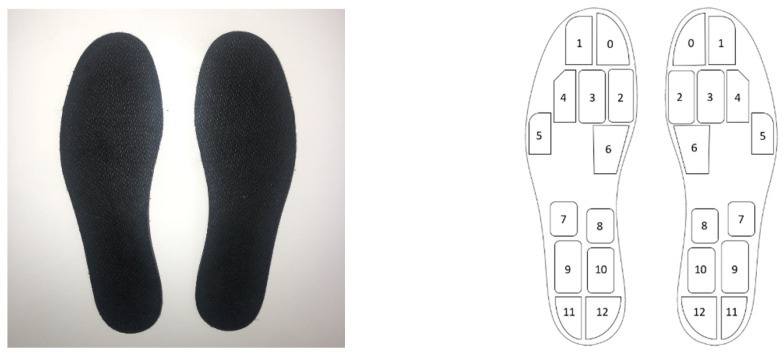
Moticon insoles and the number of sensors covering the insole area (not to scale).

The Moticon system measures left and right plantar pressure distribution, 3-dimensional acceleration and calculates reaction force from plantar pressure. The system has a mass of <0.1 kg with each insole containing 13 capacitive sensors and a 3-dimensional MEMS accelerometer (Bosch Sensortech BMA150) covering 52% of the insole area (Figure 2). Moticon insoles have a sampling frequency of 50 Hz. Each insole has 16 MB flash memory and a wireless module for data transmission. The Moticon insoles are factory calibrated with homogeneously distributed loads, covering the specified load range from 0 to 400 kPa. Moticon states that no further calibration is needed within the specified lifetime of 100 km running. Insoles were zeroed prior to each trial by completely unloading each insole as per Moticon software (01.11.00_11072-929d380, Moticon Science, Germany) guidelines [28].

**Figure 3:**
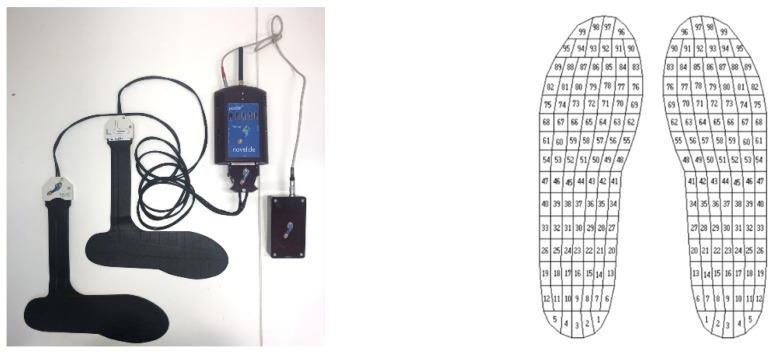
Pedar-x insole system set-up and the number of sensors covering the insole area.

The Pedar-x insole system measures left and right pressure distribution and calculates reaction force and temporal-spatial variables. The system has a mass of 0.36 kg, with each insole containing 99 embedded capacitive sensors covering 100% of the insole area (Figure 3). The Pedar-x insoles sample at 50 Hz, with a pressure measurement range of 15–1200 kPa. The Pedar hardware and Pedar-x Expert (22.3.3, Novel GmbH, München, Germany) software was used to collect the data. Pedar-x insoles were calibrated by the manufacture prior to testing. Insoles were configured (configuration allows for the software to identify calibration files) and zeroed prior to each test by unloading each insole, so there was no load on the insoles [29].

**Figure 4:**
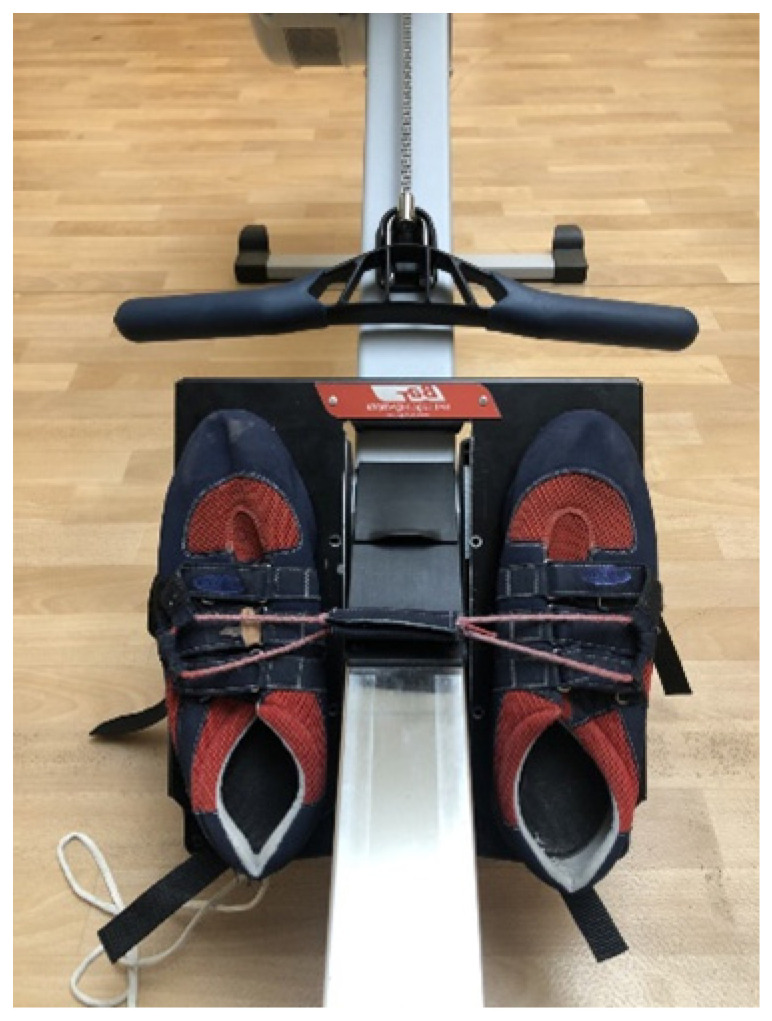
BAT Logic plate inserted onto a Concept2 Ergometer.

A footplate (ErgAdaptor, BAT Logic, Victoria, Australia) with rowing shoes (New Wave, Oberaudorf, Germany) was installed onto the Concept2 ergometer foot-stretcher (Figure 4). The foot cradle on the Concept2 was removed and the ErgAdaptor footplate was adapted to allow it to be screwed in place of the Concept2 foot-cradle.

### 2.4. Protocol

Participants had their height, mass, and foot-lengths measured using Harpenden stadiometer (Harpenden, Holtain Limited, Crymych, UK), Tanita scales (Tanita Australia, Kewdale, Australia), and long bone calipers (Harpenden, UK), respectively. The appropriate insole size was selected based on the participant’s foot-length (left foot length, 25.2 ± 1.0 cm, right foot length, 25.1 ± 0.9 cm). The resistance on the ergometer was self-selected by each athlete as per the national guidelines from Rowing Australia [30] (drag factor, 109 ± 30) and remained unchanged across each of the four trials. Participants performed a standardised warm-up of ergometer rowing (2000 m at stroke rate [SR] 18–20 strokes/min) (time to complete 2000 m 9:32.5 ± 1:18.8 min; SR, 20.2 ± 1.1 strokes/min). A rest period of 2 min was provided after the warm-up, while a pair of insoles was zeroed and then placed into the selected footwear ready for the first time-trial. Participants then performed four 500 m trials of ergometer rowing at a fixed SR of 22–24 strokes/min. The insole order (Moticon or Pedar-x) was randomly allocated for each trial, with each insole being tested twice (Figure 1). The PM5 ergometer display was partially covered so only SR and distance (m) were visible to the participants during the time trial; elapsed time, time/500 m, and power (W) were hidden. After completing each 500 m trial, participants had a 5 min rest period, which included changing (if required) and re-zeroing of the insoles. Following the completion of all trials, a standardised cool-down (1000 m at SR 18–20 strokes/min) was completed.

### 2.5. Data Analysis

Data were exported from Moticon Science software and Pedar-x Expert software as raw data and processed through Microsoft Excel 2018 (Microsoft Corporation, USA). The first and final strokes were removed from each trial. Data for left (L) and right (R) were analysed individually and combined to provide total foot values (T; left + right). Mean and peak values of plantar pressure (kPa) and reaction force (N) were calculated. Performance variables from the PM5 were exported via the FloatPro mobile application as a csv file. For each 500 m effort, effort time and average stroke rate were determined, and the following performance variables were calculated: power average (W), power max (W), drive length average (m), drive time average (s), stroke recovery time average (s), stroke distance average (m), drive force average (N), drive force max (N) and work per stroke average (J).

### 2.6. Statistical Analysis

All statistics were conducted using SPSS statistical software (v25, IBM Corp, Armonk, NY, USA). Means and standard deviations (SD) from each trial were calculated. Boxplots were used to identify outliers (no outliers were found) and the Shapiro–Wilk test was used to check data normality. If a true outlier was found after data check, the sample was removed from the analysis. Data were analysed in raw and natural logarithm transformed forms [31]. Data are reported as mean ± 95% confidence interval (CI). Reliability of Moticon and Pedar-x (Moticon 1 vs. Moticon 2; Pedar-x 1 vs. Pedar-x 2) was assessed via intraclass correlation coefficient (ICC_2,1_) (<0.5, poor; 0.5–0.75, moderate; 0.75–0.9, good; >0.9, excellent) [32], paired t-test to determine significant difference of the bias between measures (with post hoc Bonferroni correction), typical error of the estimate (TE), coefficient variation percentage (CV%) and effect size (ES)—Cohen’s d (<0.2, trivial; 0.2–0.6, small; 0.6–1.2, moderate; 1.2–2.0, large; and >2.0, very large) [33]. For the purpose of interpretation, typical error (TE) is reported in raw units (kPa or N) and as a percent (%CV). Reliability of performance variables across four trials was assessed via ICC_2,1_ and repeated measures ANOVA. To assess validity, the two tests per insole were treated as individual assessments (pooled data) which increased the sample size to N = 38. Validity between Moticon and Pedar-x insoles was assessed via paired t-test to determine the significance of the bias between measures (Moticon vs. Pedar-x), Pearson correlation (r-values were assessed as follows: 0.0–0.1, trivial; 0.1–0.3, small; 0.3–0.5, moderate; 0.5–0.7, large; 0.7–0.9, very large; 0.9–1.0, nearly perfect) [33], 95% LoA [34], standard error of mean (SEM), TE, CV%, and ES-Cohen’s d. Significance level for all *p*-value hypothesis testing was set at *p* < 0.05.

## 3. Results

All participants completed the full testing protocol. The average SR for each test was 22.3 ± 0.7 strokes/min and the average time-trial time was 2:06.7 ± 0:15.4 min. ICCs for all performance variables (power average, power max, drive length average, drive time average, stroke recovery time average, stroke distance average, drive force average, drive force max and work per stroke average) showed good-excellent reliability (ICC > 0.86) and repeated measures analysis revealed no significant difference between trials (*p* = 0.19–0.74). As such, it was considered that all four trials were performed consistently, and thus any differences identified between and within Moticon and Pedar-x insoles were not the result of differences in rowing performance.

### 3.1. Reliability

Reliability statistics are presented in Table 1. ICCs for Moticon insoles were moderate-excellent (0.57–0.92) for all pressure and force variables, demonstrating good absolute agreement. ICCs for Pedar-x insoles also showed moderate-excellent reliability (0.61–0.90) for all pressure and force variables, except for right foot peak force (F_peak_) (ICC = 0.39). Paired sample t-test revealed no significant difference (*p* > 0.11) between repeat efforts for all pressure and force variables. Effect sizes for all pressure and force variables for Moticon (ES = 0.04–0.29) and Pedar-x (ES = 0.03–0.26) were trivial-small.

### 3.2. Validity

Validity statistics are presented in Table 2. A paired t-test identified significant differences (*p* < 0.001) between Moticon and Pedar-x for average pressure (P_av_), average force (F_av_), and F_peak_. Effect size shows a large bias (ES > 1.13) and Pearson’s correlation shows small-trivial association (r < 0.37), and as such Moticon has poor relative and absolute agreement with Pedar-x insoles for P_av_, F_av_ and F_peak_ and. Due to the difference in the surface area of individual sensors in the Moticon and Pedar-x insoles, peak pressure (P_peak_) was not compared. F_av_ and F_peak_ were overestimated by Moticon, demonstrated by the very large bias (F_av_, mean bias (%) = 59.7–87.7%; ES = 1.88–2.28; F_peak_, mean bias (%) = 46.8–66.5%, ES = 1.98–2.24). P_av_ was also overestimated by Moticon, however, the moderate-large bias (P_av_, mean bias (%) = 29.1–52.2%; ES = 1.13–1.39) suggests to a lesser extent than force variables.

## 4. Discussion

This study aimed to determine the reliability and validity of Moticon sensor insoles against the Pedar-x sensor insole system for both plantar pressure and reaction force during ergometer rowing. Moticon and Pedar-x insoles displayed moderate to excellent test–retest reliability and all four trials demonstrated excellent test–retest reliability, and as such were consistent in effort (as evidenced by performance variables), however, there was a significant difference and large to very large bias between the Moticon and Pedar-x insoles.

Pressure and force variables measured by Moticon and Pedar-x insoles demonstrated moderate-excellent test–retest reliability (ICC = 0.57–0.92), consistent with previous studies [8,10,13,14,16]. However, to our knowledge, the reliability of Moticon insole systems have only been assessed for COP, temporal-spatial gait parameters, and force variables [13,14,16]. The current study demonstrated that pressure variables have a moderate-excellent test–retest reliability for (ICCs: P_av_ = 0.71–0.92; P_peak_ = 0.69–0.81) in ergometer rowing. However, Moticon insoles demonstrated a significant difference in the measured pressure and force variables (*p* < 0.001) and poor relative and absolute agreement (Pearson’s < 0.37, ES > 1.13) with Pedar-x insoles, therefore Moticon is not a valid pressure measuring system when compared to Pedar-x.

Validity assessment indicated there was a moderate-large overestimation of average plantar pressure and a large-very large overestimation of average and peak reaction force when compared to the Pedar-x insoles. Mean bias and LoA were consistent with previous studies [14,15]. Specifically, the mean bias (%) for CMJ max force shown by Stöggl and Martiner [15] (25–55%) was relatively consistent with the current study (46–66%). Nagahara and Morin [14] also compared Moticon insoles across multiple force platforms (consisting of 50 force plates) during sprinting and found mean biases (%) of −48.3 ± 12.3% and −43.4 ± 14.6% for F_av_ and F_peak_, respectively, which were consistent with the mean bias for force variables demonstrated in the present study. These studies, however, did not analyse pressure variables.

In rowing, the feet maintain contact with a surface (foot-stretcher) throughout the stroke (i.e., large ground contact time), and there is a steep rise and drop in force (i.e., short force-time curve) that occurs as the rower pushes against the foot-stretcher. The study supports previous literature highlighted that low validity and poor agreement between the systems was observed when there was a short force-time curve, high ground reaction forces, and higher impact forces, associated with high speed movements (i.e., sprinting) [14,15]. However, where previous literature found underestimation in force variables, as a result of short ground contact times [14,15], the current study found an overestimation of force variables due the large ground contact time, attributable to the continuous contact of the feet with the foot-stretcher throughout the stroke cycle. In addition, as the amount of applied force increased, the discrepancy between measuring systems increased (i.e., heteroscedasticity) [14,15]. Consequently, while the current study only assessed one exercise intensity, the results of Nagahara and Morin [14] and Stöggl and Martiner [15] indicate that increases in the applied force (as a means of increasing rowing intensity) would result in increases in greater differences between Moticon and Pedar-x systems.

It may be hypothesised that a sampling frequency of 50 Hz is too low to measure force variables to the level of accuracy required in rowing [14]. While Moticon can sample at 100 Hz, it cannot utilize all sensors at this sampling rate, so while the sampling frequency is greater, the number of sensors and surface area covered are lower. It is believed that for this study a sample rate of 50 Hz was enough to capture pressure and force variables, however, if the exercise intensity and stroke rate was increased, resulting in an increase in the movement speed of the rowing stroke, 50 Hz may be a limitation. Discrepancies between Moticon and Pedar-x insoles have been suggested to be attributed to differences in the response of the capacitive sensors [15] and the large difference in the number of sensors. A large number (99) of smaller sensors allows Pedar-x insoles to identify more precise measurements across the surface area of the foot. The Pedar-x sensors also cover the entirety of the insole, while the Moticon insoles measure across 52% of the insole surface area, again highlighting the specificity differences between the system.

A limitation to this study includes the inability to calibrate both insoles with the same calibration system, as such there is a comparison of two individual measurements, as opposed to a comparison with a true value. Additionally, the study only assessed reliability across two trials. Increasing the number of intra-day trials could improve the estimate of reliability, as would including inter-day trials to determine the physiological error associated with reliability. The study was also only conducted at a submaximal rowing intensity, determined by the SR. Based on heteroscedasticity observed in previous studies, it could be suggested that higher intensity efforts will show greater discrepancies between systems due to the increase in applied force and number of strokes per minute. As such, the assessment of Moticon insoles during high intensity rowing is desired. Moreover, further investigation of Moticon’s validity against force platforms used in both ergometer and on-water rowing is warranted. Lastly, further assessment of Moticon’s reliability in on-water rowing is required, as rowing technique differs between on-water and ergometer rowing

## 5. Conclusions

The Moticon and Pedar-x insoles have moderate-excellent test–retest reliability. However, compared with Pedar-x, Moticon insoles demonstrate poor relative and absolute agreement. Moticon insoles were found to overestimate the measured pressure and calculated force variables, which may relate to high applied forces, short force-time curves, and large ground contact times associated with rowing. Therefore, Moticon insoles cannot currently be used to accurately measure pressure and force variables over time. However, Moticon insoles are a practical and user-friendly system, and based on reliability results may be able to assess certain measures over multiple sessions in ergometer rowing, however this should be considered with caution. Due to Moticon’s lack of wires and battery packs, this system could be extremely beneficial for on-water rowing analysis, as the insoles would have no impact on the set-up of the rowing boat and minimal impact on the athlete and their rowing technique. In addition, Moticon insoles are more affordable to rowing programs and teams that cannot afford nor have the time or the expertise to set-up instrumented foot-stretchers with load cells on a rowing boat. As sport biomechanics research moves out of the lab and undertakes more in-field testing, technology such as Moticon could be the future practical option for on-water testing of training loads, boat setup and technique changes for rowing. Further research is necessary to validate the ability of Moticon insoles to measure pressure and force parameters over time in rowing.

## Figures and Tables

**Table 1:** Reliability of Moticon and Pedar-x insoles for plantar pressure and reaction force variables.

		N	Mean (SD)				Diff. in Mean (±95%CI)		ES-Cohen’s d		SEM		ICC _(2,1)_ (Ln) (95%CI)		TE (Raw)		TE as %CV (Ln)		Sig. (2-Tailed)	
			M		*p*		M	*p*	M	*p*	M	*p*	M	*p*	M	*p*	M	*p*	M	*p*
			1	2	1	2														
F_av_ (N)	L	19	255.1 (77.5)	241.5 (63.1)	149.5 (22.4)	145.7 (24.7)	13.7 (±18.1)	3.9 (±6.4)	0.19	0.16	8.6	3.1	0.90 (0.75–0.96)	0.85 (0.65–0.95)	26.6	9.4	11.5	7.1	0.13	0.22
	R	19	249.8 (56.4)	253.0 (45.0)	173.2 (33.1)	169.5 (31.6)	−3.2 (±20.7)	3.7 (±11.6)	−0.06	0.10	9.8	5.5	0.62 (0.24–0.83)	0.79 (0.53–0.91)	30.3	17.1	14.4	10.7	0.75	0.52
	T	19	505.0 (125.4)	494.5 (94.5)	322.7 (46.7)	315.2 (52.2)	10.5 (±28.8)	7.5 (±15.3)	0.10	0.15	13.7	7.3	0.81 (0.58–0.92)	0.82 (0.59–0.93)	42.2	22.5	11.3	7.5	0.46	0.32
F_peak_ (N)	L	19	736.3 (198.2)	688.8 (134.7)	471.4 (87.5)	462.6 (72.4)	47.5 (±42.0)	8.8 (±26.0)	0.29	0.11	20.0	12.4	0.91 (0.78–0.96)	0.82 (0.59–0.93)	61.7	38.1	7.5	8.3	0.35	0.48
	R	19	704.6 (124.6)	690.2 (82.8)	502.6 (55.6)	504.7 (88.1)	14.4 (±47.8)	−2.1 (±37.1)	0.14	−0.03	22.8	17.7	0.57 (0.18–0.81)	0.39 (−0.07–0.71)	70.2	54.4	11.2	13.5	0.54	0.91
	T	19	1440.9 (313.2)	1379.0 (193.6)	974.0 (130.9)	967.3 (138.8)	61.8 (±80.7)	6.7 (±59.8)	0.24	0.05	38.4	28.4	0.82 (0.60–0.93)	0.61 (0.22–0.83)	118.4	87.7	8.4	9.7	0.13	0.82
P_av_ (kPa)	L	19	13.8 (4.4)	13.1 (3.6)	9.0 (1.4)	9.3 (3.2)	0.8 (±0.9)	−0.3 (±1.3)	0.19	−0.15	0.4	0.6	0.92 (0.80–0.97)	0.65 (0.28–0.85)	1.4	1.9	10.5	14.5	0.11	0.57
	R	19	13.2 (3.4)	13.3 (2.7)	10.3 (1.8)	10.5 (2.1)	−0.1 (±1.1)	−0.2 (±0.8)	−0.04	−0.10	0.5	0.4	0.71 (0.38–0.88)	0.70 (0.38–0.87)	1.6	1.2	14.3	11.4	0.81	0.62
	T	19	27.0 (7.3)	26.4 (5.7)	19.3 (2.6)	19.8 (4.7)	0.6 (±1.5)	−0.5 (±1.9)	0.10	−0.14	0.7	0.9	0.85 (0.66–0.94)	0.61 (0.23–0.83)	2.2	2.8	11.0	12.3	0.40	0.56
P_peak_ (kPa)	L	19	97.2 (11.8)	98.8 (10.8)	335.3 (78.4)	322.2 (82.5)	−1.6 (±4.5)	13.0 (±30.6)	−0.14	0.16	2.1	14.6	0.69 (0.36–0.87)	0.72 (0.41–0.88)	6.5	45.0	6.8	14.6	0.47	0.38
	R	19	91.2 (13.1)	89.6 (12.7)	260.7 (94.9)	287.5 (110.3)	1.6 (±3.9)	−26.8 (±24.8)	0.12	−0.26	1.9	11.8	0.81 (0.57–0.92)	0.90 (0.76–0.96)	5.8	36.4	6.6	12.4	0.41	0.43
	T	19	188.4 (22.6)	188.4 (20.9)	595.9 (160.8)	609.7 (182.2)	0.0 (±7.3)	−13.8 (±48.4)	0.00	−0.08	3.5	23.1	0.77 (0.49–0.90)	0.86 (0.67–0.94)	10.7	71.1	5.9	11.7	0.99	0.56

M = Moticon, *p* = Pedar-x, F_av_ = average reaction force, F_peak_ = peak reaction force, P_av_ = average plantar pressure, P_peak_ = peak plantar pressure, L = left foot, R = right foot, T = total foot (L + R), SD = standard deviation, CI = confidence interval, ES = effect size, SEM = standard error of mean, ICC = intraclass correlation coefficient, Ln = log natural, TE = typical error of the estimate, CV% = coefficient of variation.

**Table 2:** Validity and agreement plantar pressure and reaction variables between Moticon insoles (practical) and Pedar-x (criterion).

		N	Mean (SD)		Diff in Mean (±95% CI)	%Bias (±95% CI) (Ln)	ES-Cohen’s d	SEM	TE	TE (as CV%) (Ln)	Pearson’s Correlation (95% CI) (Ln)	95% LoA		Sig. (2-Tailed)
			M	*p*								Raw	as % (Ln)	
F_av_ (N)	L	38	248.3 (70.1)	147.6 (23.4)	100.7 (±23.5)	87.7% (±24.5%)	2.16	11.6	23.5	17.9	0.15 (−0.18–0.45)	140.0	14.2	<0.001 *
	R	38	251.4 (50.3)	171.4 (35.0)	80.1 (±19.2)	59.7% (±17.1%)	1.88	9.5	35.3	23.7	0.10 (−0.23–0.41)	114.45	11.7	<0.001 *
	T	38	499.7 (109.6)	319.0 (49.0)	180.8 (±39.6)	72.6% (±18.4%)	2.28	19.5	49.7	17.7	0.03 (−0.29–0.34)	236.0	10.2	<0.001 *
F_peak_ (N)	L	38	712.6 (168.9)	467.0 (79.3)	245.6 (±53.3)	66.5% (±14.1%)	1.98	26.3	76.3	18.3	0.37 (0.05–0.61)	317.9	7.8	<0.001 *
	R	38	697.4 (104.6)	503.7 (72.7)	193.7 (±37.6)	46.8% (±10.6%)	2.19	18.5	72.1	16.8	0.21 (−0.12–0.49)	224.0	6.7	<0.001 *
	T	38	1410.0 (258.8)	970.7 (133.1)	439.3 (±84.9)	55.8% (±10.9%)	2.24	41.9	130.3	14.7	0.30 (−0.02–0.57)	506.2	5.7	<0.001 *
P_av_ (kPa)	L	38	13.4 (4.0)	9.1 (2.5)	4.3 (±1.4)	55.2% (±21.1%)	1.34	0.7	2.5	23.9	0.24 (−0.09–0.52)	8.5	35.5	<0.001 *
	R	38	13.2 (3.0)	10.4 (1.9)	2.8 (±1.2)	29.1% (±14.5%)	1.13	0.6	1.9	21.1	0.06 (−0.27–0.37)	7.0	28.7	<0.001 *
	T	38	26.7 (6.5)	19.6 (3.8)	7.1 (±2.4)	40.9% (±15.9%)	1.39	1.2	3.8	19.7	0.11 (−0.22–0.42)	14.4	21.3	<0.001 *

M = Moticon, *p* = Pedar-x, F_av_ = average reaction force, F_peak_ = peak reaction force, P_av_ = average plantar pressure, L = left foot, R = right foot, T = total foot (L + R), SD = standard deviation, CI = confidence interval, Ln = log natural, ES = effect size, SEM = standard error of mean, TE = typical error of estimate, CV% = coefficient of variation, LoA = limits of agreement, * significance = *p* < 0.05.

## Data Availability

Not applicable.
